# Emigration intentionality among Tunisian interns and residents in medicine

**DOI:** 10.1192/j.eurpsy.2022.564

**Published:** 2022-09-01

**Authors:** F. Tabib, F. Guermazi, A. Zouari, M. Ben Abdallah, S. Hentati, I. Baati, J. Masmoudi

**Affiliations:** 1 UHC Hedi Chaker, Department Of Psychiatry A, sfax, Tunisia; 2 UHC Hedi Chaker, Department Of Psychiatry A, Sfax, Tunisia

**Keywords:** residents, emigration, medecine

## Abstract

**Introduction:**

Emigration is the act of leaving one’s country of nationality or habitual residence to settle in another nation. In Tunisia, this phenomenon is increasing in particular for doctors.

**Objectives:**

Evaluating the intentionality of emigration among interns and medical residents in Tunisia while studying the factors related to it.

**Methods:**

We conducted a cross-sectional, descriptive and analytical study of interns and medical residents who participated in our study through the social network ’Facebook’ by an anonymous self-questionnaire. The level of satisfaction with the different aspects of life were assessed by a 5-point Likert scale, from “not at all satisfied” to “very satisfied”.

**Results:**

The total number of participants was 56 of which 64.3% were medical residents. More than 50% of the participants expressed dissatisfaction with the distribution of tasks and organization of work (66.1%), safety at work (53.6%), comfort (57.2%), time allocated to personal life (53.6%) and salary (69.6%). The political, health and educational situation in the country was considered unsatisfactory by the majority of participants (90% to 95%). Among our participants, 44.6% regretted having chosen the profession of medicine and 53.6% had plans to immigrate to work abroad. The intentionality of immigration was significantly higher among men (p=0.02), those with siblings abroad (p=0.047) and those without dependent relatives (p=0.040).

**Conclusions:**

Young physicians are strongly looking for emigration. This decision could emanate from professional, personal and political factors. Further studies seem to be necessary to explain this emigration phenomenon.

**Disclosure:**

No significant relationships.

